# Necrostatin-1 Attenuates Inflammatory Response and Improves Cognitive Function in Chronic Ischemic Stroke Mice

**DOI:** 10.3390/medicines3030016

**Published:** 2016-07-01

**Authors:** Shehong Zhang, Yuyang Wang, Dake Li, Junfa Wu, Wen Si, Yi Wu

**Affiliations:** 1 Department of Rehabilitation, Huashan Hospital, Fudan University, Shanghai 200040, China; zhangshehong001@163.com (S.Z.); tonywyy@163.com (Y.W.); junfawu2002@aliyun.com (J.W.); siwen@fudan.edu.cn (W.S.); 2 Department of Neurology, Huashan Hospital, Fudan University, Shanghai 200040, China; lidake@163.com

**Keywords:** necroptosis, ischemia, cognition, inflammation

## Abstract

Multiple cell death is involved in ischemic brain injury. Necroptosis, a recently reported cell death, may be the most suitable cell death mechanism in a subpopulation of neurons under ischemic injury. It reported that a small molecule, necrostatin-1 (Nec-1), has a potent inhibitory effect on necroptotic cell death in vivo and in vitro. The aim of the current study was to investigate the role of Nec-1 on cognitive function in chronic ischemic stroke mice induced by bilateral common carotid artery stenosis (BCAS). Here, 12-week-old C57BL/6 mice received intragastric administration with Nec-1 or vehicle for two weeks after stroke, and then, the effect and possible mechanism were determined. We demonstrated that inhibition of necroptosis prevented cognitive impairment and reduced inflammatory response in the ischemic brain injury mouse model. These data suggested that inhibition of necroptosis provided a potential therapeutic option for cognitive rehabilitation in chronic ischemic stroke.

## 1. Introduction

Ischemic stroke accounts for about 80% of all cases of stroke [1] and frequently leads to cognitive dysfunction [2]. Cognitive dysfunction induced by reduced blood supply to the brain has been termed “vascular cognitive impairment” (VCI) [2,3,4] and it has become a significant healthcare concern. Experimental studies demonstrated that white matter lesions (MWLs) from ischemia are the main factor mediating the pathological process of VCI [5,6,7].

Moreover, white matter ischemic injury is a critical pathological hallmark of small vessel diseases, a subset of cerebrovascular diseases resulting in stroke and cognitive decline [8]. Experimental and clinical studies demonstrated that a persistent and chronic reduction in cerebral blood flow leading to hypoperfusion resulted in white matter ischemic damage [5,9,10]. WM alternations are regulated by many factors and multiple processes, such as vascular dysfunction and inflammation, blood-brain barrier (BBB) disruption, glial activation, damaged oligodendrocytes, and demyelination [5].

It was reported that inflammatory activation and sustained microvascular failure are major mediators of stroke and cerebral ischemia [11]. A recent study showed that the condition created by the ischemic injury is suitable for necroptosis, a programmed necrosis, that can be inhibited by Nec-1 [12], which triggers an inflammatory response. Under the condition of chronic cerebral hypoperfusion, durative inflammation may result in regional perfusion deficits and deficient blood flow sustained at relative white matter tracts. Therefore, we hypothesized that inhibition of necroptosis after ischemic brain injury should alleviate inflammation and improve cognitive function in the bilateral common carotid artery stenosis (BCAS) mouse model.

## 2. Results and Discussion

### 2.1. Physiological Index and Mortality Rates in the Experimental Animals

**Figure 1:**
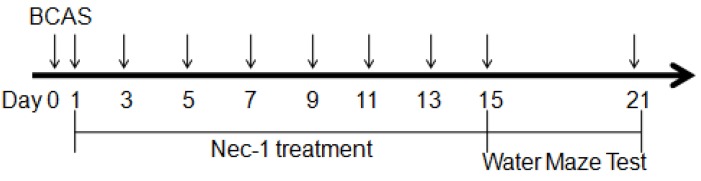
The diagram of the experimental design. Mice underwent surgery for BCAS at day 0 and were treated with Nec-1 every other day after surgery until the 15th day.

**Figure 2:**
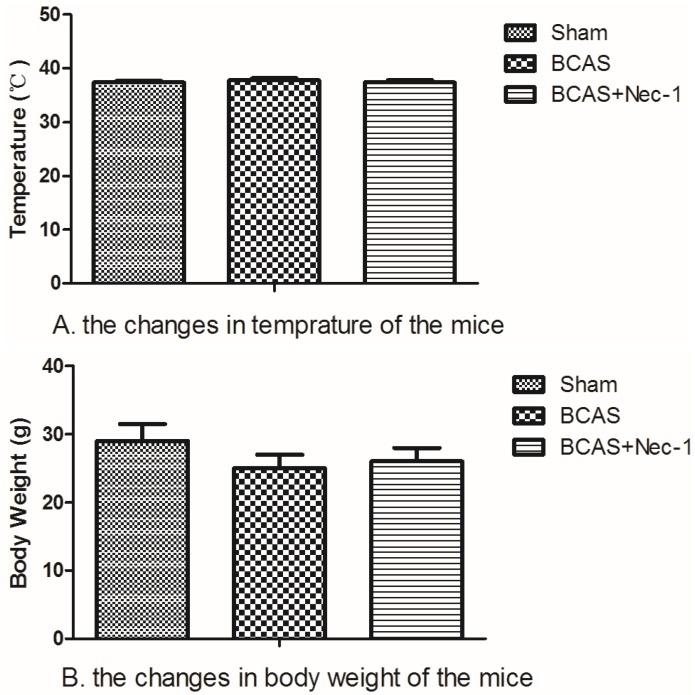
The changes in temperature and body weight of the mice. (**A**) The changes in temperature of the mice were assayed. At the 15th day, temperature of the mice had no statistical difference in three groups (*P* > 0.05); (**B**) The changes in body weight of the mice were assayed. At the 15th day, temperature of the mice had no statistical difference in three groups (*P* > 0.05), although the body weight of the mice has a downward trend in the BCAS and BCAS with Nec-1 groups.

**Figure 3:**
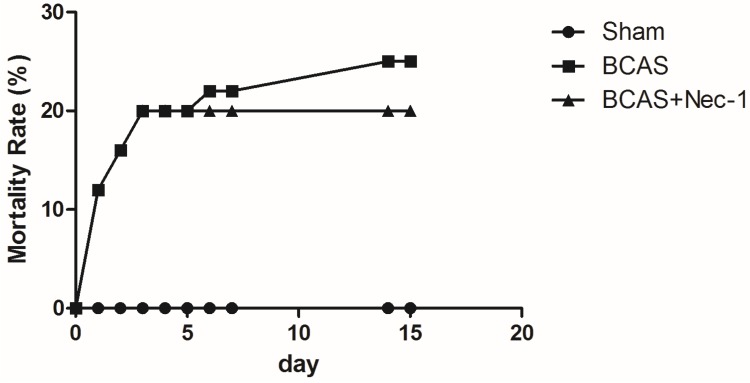
The mortality rate of the mice after surgery. The death of the mice occurred mainly in the first three days after surgery. The mortality rate in the BCAS group was lower than in the BCAS with Nec-1 group, but the difference was not statistically significant.

The number of animals used in this study and the whole experimental design is displayed in Figure 1. All procedures for BCAS were accomplished in no more than 15 min. The temperature of the surviving mice did not change significantly among the three groups at the 15th day. The body weight decreased after the surgery but recovered to the baseline at the 15th day in all groups. Although the mice in both BCAS (*n* = 15) and BCAS (*n* = 15) with Nec-1 groups tended to have a lower body weight than in the sham group (*n* = 15), the results had no statistical significance (Figure 2). After surgery, all of the animals recovered within several hours but the mental state was still not good, including symptoms such as listlessness and loss of appetite. None of them showed any apparent motor weakness. The death of the mice occurred mainly in the first three days after surgery (Figure 3).

### 2.2. Changes of Cerebral Blood Flow (CBF) in the Experimental Mice

**Figure 4:**
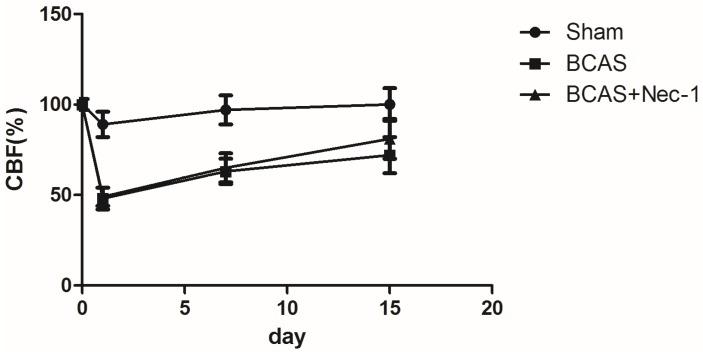
The values of CBF in the mice. There was a sharp reduction of the CBF in the BCAS group 2 h after surgery and then it increased gradually. Although there was a lower CBF in the two BCAS operation groups than in the sham group, the change was not statistically significant.

In Figure 4, data showed the mean values of CBF in the surviving mice. In the sham group, the mean values of CBF after the operation have no significant changes between any time intervals. In contrast, the CBF values decreased significantly between the preoperative baseline and 2 h after the surgery in the BCAS group, and we observed a sharp reduction of the CBF values to (50 ± 8)%. At the 15th day, the CBF values in the BCAS and BCAS with Nec-1 groups were still lower than in the sham group. Intergroup differences in the CBF values were also detected, but no significant differences were noted among the three groups.

### 2.3. Nec-1 Improves Cognitive Decline in the BCAS Mice

**Figure 5:**
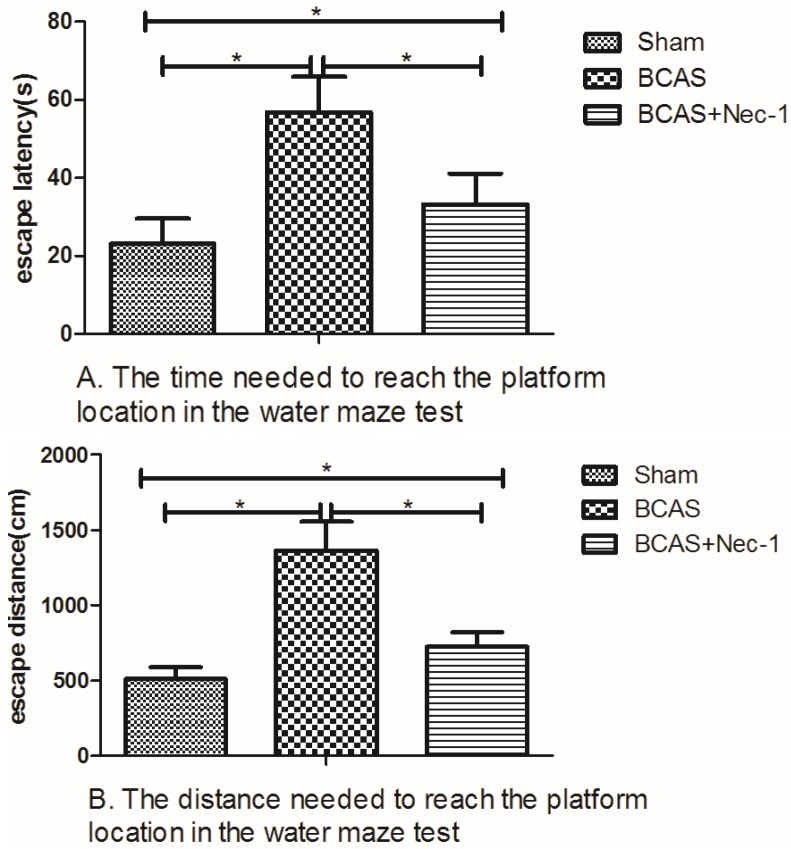
Nec-1 improved cognitive function in the mice. (**A**) The time and distance needed to reach the platform location in the water maze test. Data showed that the time in the BCAS group was longer than in the BCAS with Nec-1 group (* *p* < 0.05); (**B**) The distance needed to reach the platform location in the water maze test. Data showed that the distance in the BCAS group was longer than in the BCAS with Nec-1 group (* *p* < 0.05).

As shown in Figure 5, there was a statistical difference in the effect of Nec-1 on the cognitive function of BCAS mice, including escape latency and escape distance.

The changes of escape latency showed statistical significance among the sham (23.12 ± 6.52), BCAS (56.78 ± 9.14), and BCAS with Nec-1 (33.24 ± 7.86) groups. The BCAS group showed increased escape latency compared to the sham group and the BCAS with Nec-1 group (*p* < 0.05). The results of the escape distance also showed statistical differences among the sham (511.54 ± 78.17), BCAS (1365.43 ± 190.01), and BCAS with Nec-1 (723.55 ± 96.79) groups. The BCAS group showed increased escape distance compared to the other two groups (*p* < 0.05).

### 2.4. Nec-1 Decreased the Expression of Proteins from Hippocampus

**Figure 6:**
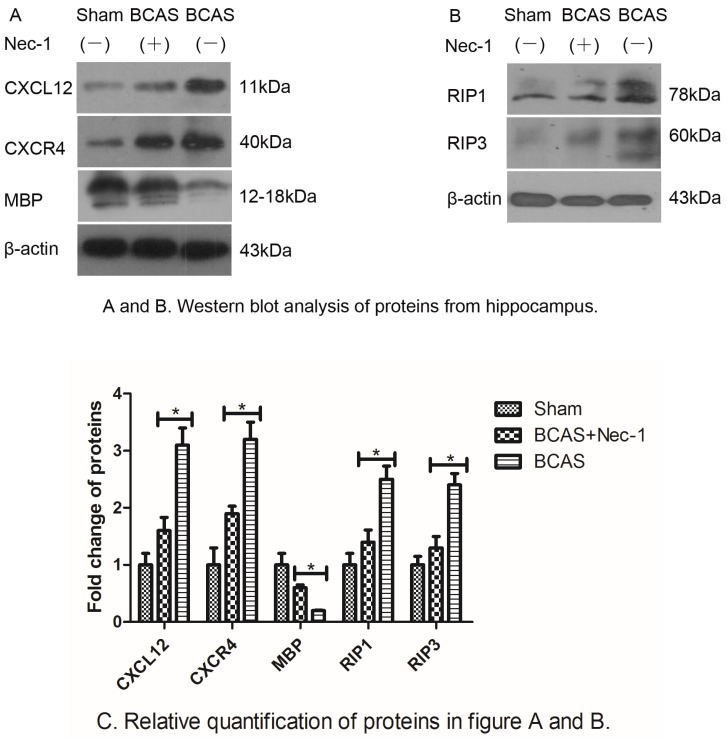
Western blot analysis of proteins from hippocampus. (**A**) The level of CXCL12, CXCR4, MBP and β-actin proteins. The mice in the BCAS group had significantly higher levels of CXCL12 and CXCR4 proteins, but lower level of MBP protein than in the BCAS with Nec-1 group (* *p* < 0.05); (**B**) The level of RIP1, RIP3 and β-actin proteins. The mice in the BCAS group had significantly higher levels of RIP1 and RIP3 proteins than in the BCAS with Nec-1 group (* *p* < 0.05).

To understand how Nec-1 improved cognitive function of the mice, we examined myelin basic protein (MBP) expression to assess the myelin lesion using Western blot. Myelin is a major component of white matter and is more sensitive to the ischemic injury, and the loss of myelin influences cognitive performance. The results suggested that Nec-1 inhibited the decline of the MBP protein. At the same time, we examined the expressions of chemokines and necroptosis-related proteins, such as CXCL12, CXCR4, RIP1 and RIP3. In Figure 6, data demonstrated that all these proteins decreased after Nec-1 treatment. 

### 2.5. Inflammatory Cytokine Protein and mRNA Levels were Reduced by Nec-1

**Figure 7:**
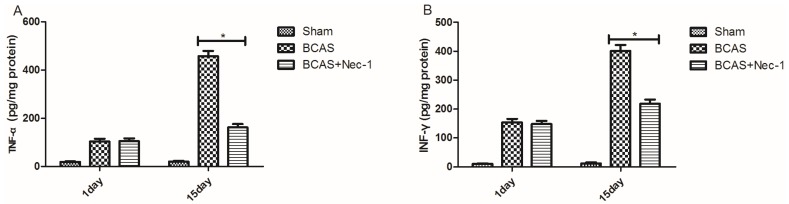

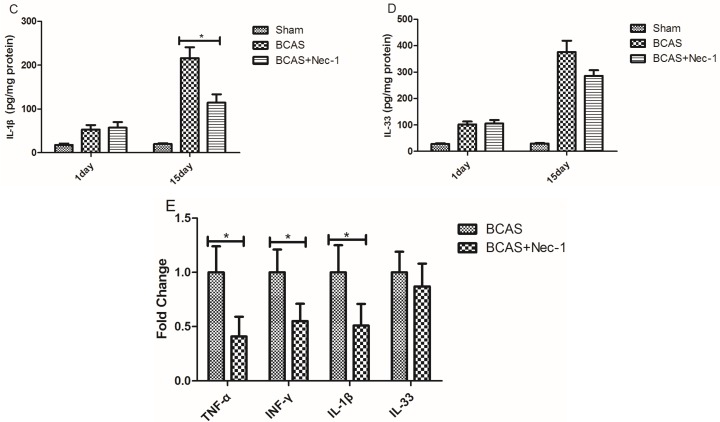
Inflammatory cytokines expression was decreased in the mice treated with Nec-1. Data showed that the protein concentrations of TNF-α (**A**), IFN-γ (**B**), IL-1β (**C**) and IL-33 (**D**) in brain tissues at the 15th day after BCAS surgery as measured by ELISA and that mRNA levels of these cytokines had the same change (**E**) (* *p* < 0.05).

RT-PCR and ELISA were used to evaluate the changes of inflammatory cytokines in protein and mRNA levels. In the Figure 7, there was a down-regulation of TNF-α, INF-γ, IL-1β and IL-33 protein expression at the 15th day after Nec-1 treatment (A–D). Nec-1 reduced TNF-α, INF-γ, IL-1β and IL-33 mRNA expression at the 15th day after BCAS (E).

### 2.6. Discussion

In the current experiment, we used Nec-1, a potent and specific small-molecule inhibitor of necroptosis, to treat the experimental mice. The study, reported here, showed that Nec-1 leads to a significant improvement in cognitive performance in the Morris water maze test and to a decrease in the expression of inflammatory markers. Furthermore, there was an increase in the MBP protein, a marker of myelin, after treatment with Nec-1.

Stroke is a devastating neurological disease with limited functional recovery. In fact, subcortical white matter stroke constitutes 15% to 25% of all stroke subtypes [13,14]. A number of studies have shown that permanent BCAS contributed to chronic ischemic white matter stroke and then induced cognitive impairments in the Morris water maze test [15,16,17,18]. Some prospective studies showed that white matter was vulnerable to the reduction of cerebral blood flow [10,19] and that small white matter strokes were associated with progressive cognitive decline [20]. The studies reported previously suggested that Nec-1 affected the kinetics of the dilation of peritubular capillaries after treatment of radiocontrast media (RCM) and that RIPK3-deficient mice showed increased renal perfusion in ischemia-reperfusion injury (IRI) models [21,22]. Therefore, the protective effects of nec-1 in chronic ischemic injury models may be involved in the mechanism of increase of blood flow due to dilation of capillaries, which needs to be further studied.

To determine myelin alterations, we performed western blot for the MBP protein. There was a marked reduction of MBP protein in the BCAS and BCAS with Nec-1 groups compared to the sham group. At the same time, we observed that the MBP protein was increased in the Nec-1 treatment group compared with the vehicle treatment group and that cognitive performance in the water maze test was improved in the Nec-1 treatment group. The study suggested that Nec-1 could also protect against ferroptosis through an unknown target [23]; more specifically, the Nec1-derivative Nec1s did not protect against Gpx4-depletion-induced cell death [23,24] and ferroptosis was involved in rat brains [25]. Therefore, the use of Nec-1s would be certainly superior in order to avoid the influence on ferroptosis which is the limitation of the current study and needs to be further researched.

Necrotic cell death is a common phenomenon in a wide variety of pathological conditions [23,24,25,26], including ischemic stroke [27,28]. However, little attention has been paid to develop therapeutics to specifically target necrosis, because of the conventional idea that necrotic cell death is an unregulated response to overwhelming stress. Interestingly, a number of studies in recent years demonstrated that physiological and pathological necrosis could be elicited in a regulated pattern [29], now named as “programmed necrosis” or “necroptosis”, differing from passive necrosis [30,31,32]. White matter of the mammalian central nervous system suffers irreversible injury when subjected to anoxia/ischemia [33,34] and ischemic brain injury may provide conditions that are suitable not for apoptosis but for necroptosis [12]. Consistently, in the study, we observed that Nec-1 significantly decreased the induction of RIP1 and RIP3 proteins, two crucial markers of necrotosis, in a BCAS mouse model.

To determine the role of inflammation following chronic ischemic stroke, some crucial proinflammatory cytokines, such as TNF-α, IFN-γ, IL-1β, IL-33, CXCL12, CXCR4, have been detected using RT-PCR and ELISA and Western blot technologies, respectively. Evidence suggested that all these proinflammatory cytokines were reduced in the Nec-1 treatment group via the vehicle treatment group, which was consistent with the previous lectures reporting that several proinflammatory cytokines, including IFN-γ, TNF-α, IL-1β, IL-2, and IL-6, have important roles in perinatal white matter injury [35,36].

Our results demonstrated that Nec-1 improved cognitive performance and reduced inflammatory response after chronic hypoperfusion, which provided an important experimental basis and therapeutic method to the clinic therapies for chronic ischemic stroke. However, some mechanisms are still unclear, such as the mechanism of the change of CXCL12 and CXCR4. So, we next investigate the serial changes in white matter and microvasculars of the brain in the ischemic stroke in depth, which should have important clinical implications and social value.

## 3. Experimental Section

### 3.1. Animals and Experimental Design

Male wild-type C57BL/6 mice were purchased from the Shanghai Laboratory Animal Center (SLAC), Chinese Academy of Sciences. Animal protocol was approved by the Institutional Animal Care and Use Committee of Fudan University, Shanghai, China, (20160966A284, 27 February 2016). Animals were provided with free access to abundant food and water. Furthermore, all animals were housed in accredited facility of 12/12 h light/dark cycle. Studies were performed on mice 12 weeks old (weighing 29 ± 2 g) during the 9 a.m.–5 p.m. time period. Mice were randomly divided into three groups: sham-operated group for procedures of BCAS and therapy (sham group, *n* = 15), BCAS group (vehicle treatment, *n* = 15), and BCAS with Nec-1 treatment group (*n* = 15).

### 3.2. Establishment of Mouse Model of Bilateral Carotid Artery Stenosis Procedure (BCAS)

The surgery of bilateral carotid artery stenosis (BCAS) was performed using microcoils made of piano wire characterized by inner diameter of 0.18 mm, wire diameter of 0.08 mm, pitch of 0.50 mm, and total length of 2.5 mm (Sawane Spring Co., Wuxi, China), according to previous publications [18,37,38,39]. Briefly, 12-week-old mice were anesthetized with 4% chloral hydrate (0.05 mL/10 g). Cerebral blood flow (CBF) was recorded through exposed skull in the prone position by laser Doppler flowmetry (Omega Wave, Tokyo, Japan) before the surgery. Then, the mouse was fixed in the supine position. Through a midline incision in the cervical part, left and right common carotid arteries were exposed in order to make microcoils applied. The body temperature was maintained at 37.0 ± 0.5 °C by a heating lamp during the course of the surgery. And then, the baseline CBF recordings were measured at one, seven, and 15 days after the surgery. The CBF values were described in a percentage of the baseline value.

On the second day after surgery for BCAS, survived animals were randomly divided into two groups which received treatment of Nec-1 or vehicle. The sham group was given the same operation without BCAS. All the animals were euthanized at the 21st day after surgery. The body weight and temperature were monitored two times a week until euthanized.

### 3.3. Drug Treatment

Mice were given either vehicle or 3.5 mg/kg Necrostatin-1 (Calbiochem, San Diego, CA, USA). For drug administration, we dissolved Nec-1 in 10% methyl-β-cyclodextrin solution (Sigma–Aldrich, St, Louis, MO, USA) and performed intragastric administration at the time points indicated. 

### 3.4. Water Maze Testing

To determine spatial learning and memory, animals were performed water maze test as described previously study reported by our team with blind method [40]. Water maze test was commenced after two weeks treated with Nec-1 or vehicle. In brief, the protocol is as follows. Water was poured into a cylindrical water tank and kept at the temperature of 22–25 °C. And then edible white pigment was dissolved in water so that the target cannot be seen. The target was placed in the water tank floor about 1 cm below the surface of the water and located in the same position during the course of experiment. Mice were trained for five days continuously and started from the different place and arrived at the target four times a day. The data was obtained and considered as experimental value only when mice found and climbed the hidden target within 60 s. On the sixth day, mice were made to start swimming while the target was removed, and time needed to reach the target place was recorded as experimental value. Each examination was performed with a minimum of 10 min interval. The percent of time spent in the target quadrant and the distance swimming were recorded as the evaluation data.

### 3.5. Sample Collection

After water maze test, the animals were anesthetized using an intraperitoneal injection of 4% chloral hydrate. After decapitation, the hemispheres were harvested for analysis.

### 3.6. Western Blot Analysis

On the 21st day after surgery, animals were deeply anesthetized with 4% chloral hydrate, and then hippocampal regions of the brain were dissected and rinsed with homogenization buffer. Total protein extracts and western blotting analysis were performed as previously described [41]. Tissues were lysed in a homogenizer containing RIPA lysis buffer keeping in 0–4 °C (Beyotime Biotechnology, Shanghai, China). Each protein sample of 40 μg was dispensed to the SDS/PAGE gel well and separated by 10%–15% SDS/PAGE and transferred onto PVDF membranes (Bio-Rad Laboratories, Hercules, CA, USA). After that membranes were blocked with 3%bovine serum albumin (BSA) and immunoblotted with primary and secondary antibodies. Protein levels were assayed of RIP1, RIP3, CXCL12, CXCR4, MBP and β-actin (Santa Cruz Biotechnology, Inc., Santa Cruz, CA, USA) respectively.

### 3.7. Enzyme-Linked Immunosorbent Assay Analysis (ELISA)

Concentrations of TNF-α, INF-γ, IL-1β and IL-33 from the hippocampal homogenates were quantified using ELISA kits (Mouse TNF-αELISA Kit; Mouse INF-γELISA Kit; Mouse IL-1βELISA Kit; Mouse IL-33ELISA Kit, RayBiotech, Norcross, GA, USA) according to the manufacturer’s protocol. Data from each sample were normalized for the protein concentration.

### 3.8. Real-Time PCR

According to manufacturer’s protocol, Total RNA from brain was isolated using TRIzol reagent (Invitrogen, Carlsbad, CA, USA) and then dissolved in 40 μL of RNase-freewater. RNA concentration was assessed by a spectrophotometer (NanoDropTM 1000A, Thermo Scientific, Wilmington, NC, USA). Amplification was performed for 40 cycles by a fast real-time PCR system using SYBR Premix ExTaq Kit (TaKaRa, Shanghai, China). A universal two-step RT-PCR cycling condition was used: 95 °C for 30 s followed by 40 cycles of 95 °C for 5 s and 60 °C for 30 s. Relative mRNA expression levels were normalized to the endogenous control, GAPDH expression, in triplicate and calculated using fold change relative to the control group [42]. Primers (Invitrogen, Shanghai, China) were synthesized as follows: TNF-α: Forward-5’-CGTCAGCCGATTTGCTATCT-3’; Reverse-5’-CGGACTCCGCAAAGTCTAAG-3’; IFN-γ: Forward-5’-GCCACGGCACAGTCATTGA-3’; Reverse-5’TGCTGATGGCCTGATTGTCTT-3’; IL-1β: Forward-5’-GCCCATCCTCTGTGACTCAT-3’; Reverse-5’-AGGCCACAGGTATTTTGTCG-3’; IL-33: Forward-5’-CACCCCTCAAATGAATCAGG-3’; Reverse-5’-GGAGCTCCACAGAGTGTTCC-3’; GAPDH: Forward-5’-AGGTCGGTGTGAACGGATTTG-3’; Reverse-5’-GGGGTCGTTGATGGCAACA-3’.

### 3.9. Statistical Analysis

Results were presented as mean ± SD. Statistical significance was set at a level of 0.05. All statistical analyses were conducted using the Statistical Package for the Social Sciences (SPSS 20.0, SPSS Inc., Chicago, IL, USA). All graphs were constructed in GraphPad Prism (version 5, GraphPad Software, San Diego, CA, USA). For comparison between the two groups or multiple groups, statistical significance was determined through a Student's t-test and one-way ANOVA followed by a Student-Newman-Keuls test, respectively.

## 4. Conclusions

The present results demonstrated, for the first time, that Nec-1 improved cognitive performance of the mice with chronic cerebral hypoperfusion induced by BCAS. This study will be helpful for clarifying the neuroprotection mechanisms of Nec-1 by inhibiting the necroptosis pathway and regulating inflammatory cytokines and is essential to the effective clinical treatment of chronic ischemic stroke.
